# Effects of the Partial M_1_ Muscarinic Cholinergic Receptor Agonist CDD-0102A on Stereotyped Motor Behaviors and Reversal Learning in the BTBR Mouse Model of Autism

**DOI:** 10.1093/ijnp/pyab079

**Published:** 2021-11-16

**Authors:** Onella Athnaiel, Greeshma A Job, Roberto Ocampo, Pamela Teneqexhi, William S Messer, Michael E Ragozzino

**Affiliations:** 1 Department of Psychology, University of Illinois, Chicago, Illinois, USA; 2 Laboratory of Integrative Neuroscience, University of Illinois, Chicago, Illinois, USA; 3 Departments of Pharmacology and Experimental Therapeutics, and Medicinal and Biological Chemistry, University of Toledo, Toledo, Ohio, USA

**Keywords:** Autism, behaviors, BTBR, learning, repetitive muscarinic receptor

## Abstract

**Background:**

Autism spectrum disorders (ASD) are a set of neurodevelopmental disorders marked by a lack of social interaction, restrictive interests, and repetitive behaviors. There is a paucity of pharmacological treatments to reduce core ASD symptoms. Various lines of evidence indicate that reduced brain muscarinic cholinergic receptor activity may contribute to an ASD phenotype.

**Methods:**

The present experiments examined whether the partial M_1_ muscarinic receptor agonist, 5-(3-ethyl-1,2,4-oxadiazol-5-yl)-1,4,5,6-tetrahydropyrimidine hydrochloride (CDD-0102A), alleviates behavioral flexibility deficits and/or stereotyped motor behaviors in the BTBR mouse model of autism. Behavioral flexibility was tested using a reversal learning test. Stereotyped motor behaviors were measured by eliciting digging behavior after removal of nesting material in a home cage and by measuring repetitive grooming.

**Results:**

CDD-0102A (0.2 and 0.6 mg/kg but not 1.2 mg/kg) injected prior to reversal learning attenuated a deficit in BTBR mice but did not affect performance in B6 mice. Acute CDD-0102A treatment (1.2 and 3 mg/kg) reduced self-grooming in BTBR mice and reduced digging behavior in B6 and BTBR mice. The M_1_ muscarinic receptor antagonist VU0255035 (3 mg/kg) blocked the effect of CDD-0102A on grooming behavior. Chronic treatment with CDD-0102A (1.2 mg/kg) attenuated self-grooming and digging behavior in BTBR mice. Direct CDD-0102A infusions (1 µg) into the dorsal striatum reduced elevated digging behavior in BTBR mice. In contrast, CDD-0102A injections in the frontal cortex were not effective.

**Conclusions:**

The results suggest that treatment with a partial M_1_ muscarinic receptor agonist may reduce repetitive behaviors and restricted interests in autism in part by stimulating striatal M_1_ muscarinic receptors.

**Significance Statement**

Despite extensive research to understand the pathophysiology of autism spectrum disorders (ASD) there has been limited translation of preclinical findings into effective treatments. Increasing evidence suggests that reduced brain muscarinic cholinergic receptor activity may contribute to an ASD phenotype. The current study determined whether the partial M_1_ muscarinic receptor agonist, CDD-0102A, alleviates a behavioral flexibility deficit and/or stereotyped motor behaviors in the BTBR mouse model of autism. Acute treatment with CDD-0102A attenuated a behavioral flexibility deficit in BTBR mice. Acute and chronic treatment with CDD-0102A attenuated an elevation in repetitive motor behaviors associated with a change in the home environment and self-grooming behavior in BTBR mice. Direct infusions of CDD-0102A into the dorsal striatum, a brain area that is altered in autism, also reduced repetitive motor behaviors in BTBR mice. The results suggest that treatment with a partial M_1_ muscarinic receptor agonist may reduce both behavioral flexibility and repetitive motor behaviors in autism.

## Introduction

Restricted, repetitive behaviors (RRBs), along with persistent reductions in social communication and interaction, represent the defining features in autism (American Psychiatric Association 2013). RRBs in autism spectrum disorder (ASD) refer to stereotyped motor actions, for example, hand flapping and sensory manipulation of objects, as well as insistence of sameness features that include circumscribed interests, rituals, and compulsions (Szatmari et al., 2006; Lam et al., 2008). A recent meta-analysis revealed that pharmacological treatments have limited effects in reducing RRBs, with anti-psychotics showing modest effects (Zhou et al., 2020), although these treatments also have negative side effects (Hesapcioglu et al., 2020).

Pharmacological treatments for RRBs in ASD commonly target the dopaminergic and/or serotonergic systems (Zhou et al., 2020). Treatments that target cholinergic systems may provide an alternative therapeutic approach while concomitantly minimizing negative side effects. Targeting the cholinergic system may be beneficial because post-mortem results from ASD individuals indicating reduced expression of both muscarinic and nicotinic cholinergic receptors in the brain (Perry et al., 2001). Furthermore, gene networks that confer risk for ASD include genes related to cholinergic transmission (Lee et al., 2012). In rodent studies, an acetylcholinesterase inhibitor, administered either systemically or injected into the dorsomedial striatum, reduces behavioral inflexibility and social impairments in the BTBR mouse model of autism (Karvat and Kimchi 2014). Moreover, the non-specific muscarinic cholinergic receptor agonist oxotremorine reduces stereotyped motor behaviors (marble burying and self-grooming behavior) in the BTBR mouse (Amodeo et al., 2014a). Taken together, the findings suggest that reduced brain muscarinic receptor activity may contribute to RRBs in ASD.

An approach that targets specific muscarinic receptor subtypes may be most beneficial in reducing core symptoms in ASD while limiting unwanted side effects. In past studies, our group found blockade of M_1_ muscarinic receptors impairs behavioral flexibility (McCool et al., 2008) while the partial M_1_ muscarinic receptor agonist 5-(3-ethyl-1,2,4-oxadiazol-5-yl)-1,4,5,6-tetrahydropyrimidine hydrochloride (CDD-0102A) enhances behavioral flexibility in rats (Ragozzino et al., 2012). Further, M_1_ muscarinic receptors are moderately to highly expressed in brain areas found altered in ASD such as the cortex, striatum, and hippocampus (Tice et al., 1996; Moran et al., 2019). Thus, stimulating M_1_ muscarinic receptors may be an effective therapeutic strategy for reducing RRBs.

The present studies determined whether the partial M_1_ muscarinic receptor agonist CDD-0102A attenuates a probabilistic reversal learning deficit and elevated stereotyped motor behavior in the self-grooming test and nesting removal test in BTBR mice compared with that of B6 mice. The BTBR mouse was chosen because this mouse exhibits a strikingly similar phenotype to ASD individuals when using similar spatial discrimination tests (Amodeo et al., 2012, 2017, 2018; D’Cruz et al., 2013) and exhibits stereotyped motor behaviors (Amodeo et al., 2019, 2021; Rhine et al., 2019; Ryu et al., 2021).

## MATERIALS AND METHODS

### Subjects

Male and female BTBR *T*^+^*Itpr3*^*tf*^/J (BTBR) and C57BL/6J (B6) mice originating from breeding colonies served as subjects. Mice were used in all experiments at approximately 8–12 weeks of age. Separate cohorts of mice were used in each study. No sex difference was observed in behavior, and thus each group in a study contained an approximately equal number of male and female mice. Unless noted, mice received food and water ad libitum throughout an experiment. A 12-hour-light/-dark cycle was employed throughout the study. Animal care and use was in accordance with the National Institutes of Health Guide for the Care and Use of Laboratory Animals and approved by the Institutional Laboratory Animal Care and Use Committee at the University of Illinois at Chicago.

### Reagents

The sample of CDD-0102A was provided by Dr William S. Messer Jr, from the University of Toledo. VU 0255035 was purchased from Tocris Bioscience (Ellisville, MO, USA).

#### Effect of CDD-0102A on Spatial Reversal Learning

Because there is no difference in acquisition or retention of a spatial discrimination between BTBR and B6 mice (Amodeo et al., 2012, 2014b, 2017, 2018; Yang et al., 2012), the effects of CDD-0102A were examined only in reversal learning. Training and testing was conducted in a black acrylic maze (76 cm long × 50 cm wide × 30 cm high) containing a start and choice area separated by a center wall (Amodeo et al., 2012; see supplementary Figure 1). A small plastic door (10 cm high × 5 cm wide) was inserted into the center wall separating the start and choice areas.

Mice were food restricted until reaching 85% of their ad libitum body weight. Training sessions began by placing a mouse into the start area. The start door was opened, allowing the mouse to enter the choice area. After consuming a one-half piece of Fruity Pebbles (Post Foods, St. Louis, MO, USA) from each food well, the mouse returned to the start area. This sequence was repeated until 15 minutes had elapsed. Mice achieved training criterion after completing 6 or more trials within a 15-minute session for 2 consecutive days. Mice were trained for 2–4 days before testing.

For testing, 1 food well was baited with a one-third cereal piece. One choice location was designated as the “correct” location and contained a cereal piece with 80% probability across trials. On the other 20% of trials, the “incorrect” location contained a cereal piece. If a mouse chose the “correct” location, it was allowed to eat the cereal and return to the start area. If a mouse chose the incorrect location, it was allowed to navigate to the unbaited food well in that location. Subsequently, the center door was raised, which allowed a mouse to return to the start area. After incorrect choices, the baited food well was temporarily removed to prevent a mouse from obtaining a cereal reinforcer. Between trials, the choice area was cleaned with a 2% quatricide solution to minimize the use of odor cues. The inter-trial interval was approximately 15 seconds. Learning criterion was achieved when a mouse chose the correct location on 6 consecutive trials.

Reversal learning was conducted the day after acquisition testing. Prior to reversal learning, each mouse received a retention test in which a mouse was reinforced with 80% probability for choosing the same spatial location as in acquisition. Criterion was achieved when a mouse successfully chose the correct spatial location on 5 out of 6 trials (Amodeo et al., 2017). Following the retention test, each BTBR and B6 mouse was assigned to 1 of 4 treatment groups: (1) saline, (2) CDD-0102A 0.2 mg/kg, (3) CDD-0102A 0.6 mg/kg, or (4) CDD-0102A 1.2 mg/kg. CDD-0102A was mixed in saline. Each treatment was injected i.p. at a volume of 10 mL/kg, 30 minutes prior to reversal learning. For example, a 30-g mouse would receive a volume of 0.3 mL. Doses were chosen based on a pilot study and were lower than those used in the other experiments, consistent with past work showing that lower doses of the drug are effective when rodents are under food restriction compared with ad libitum feed. The experimenter was not blind to treatment in any experiments, because CDD-0102A often produced some early post-injection defecation that was distinct from vehicle. For stereotyped motor tests, a second trained experimenter, blind to treatment, measured digging duration or grooming duration from the video recording to verify measurements from the first experimenter.

Separate mice were used for each treatment condition such that a mouse received only a single treatment (n = 12–13 per group). All aspects of reversal learning were identical to those in acquisition, except that the reinforcement contingencies were switched from those in acquisition. An error analysis was conducted to determine whether a treatment affected the ability to initially inhibit the previously relevant choice pattern (perseverative errors) and/or the ability to maintain the new choice pattern after the new correct choice is initially selected and reinforced (regressive errors). The first trial of reversal learning was not counted as a perseverative error but served as initial negative feedback. On subsequent trials, if a mouse chose the previously correct location, this was recorded as a perseverative error until a mouse first chose the new correct location. After selecting the correct location for the first time, all subsequent entries into the previously reinforced location were scored as regressive errors.

All mice achieved learning criterion. Thus, no mice were excluded from this study.

#### Effect of CDD-0102A in Self-Grooming Test

The test was conducted in an empty, clear plastic cage (28 cm wide × 17 cm long × 12 cm high). A camera mounted above the cage connected to a computer that ran ANY-maze software (Stoelting Co., Woodale, IL, USA). A mouse was placed in the empty cage and allowed to freely explore for 10 minutes to habituate. In the subsequent 10 minutes, grooming duration was measured. Grooming behavior included head washing, body grooming, genital/tail grooming, and paw and leg licking. A trained observer recorded the cumulative amount of time spent self-grooming. The ANY-maze software measured locomotor activity during a 10-minute grooming test.

Three experiments were conducted. In an acute treatment study, each B6 and BTBR mouse was assigned to 1 of the following 3 groups: (1) saline, (2) CDD-0102A 1.2 mg/kg, or (3) CDD-0102A 3 mg/kg. Separate mice were used for each treatment condition such that a mouse received only a single treatment (n = 7–8 per group). Twenty minutes after an i.p. injection, the habituation phase commenced for 10 minutes directly followed by a 10-minute self-grooming test as described above. Second, a study determined whether CDD-0102A may act principally through M_1_ muscarinic receptors by examining whether the selective M_1_ muscarinic receptor antagonist, VU0255035 blocked the effects of CDD-0102A. Each B6 and BTBR mouse was assigned to 1 of the following 4 groups: (1) saline/saline with 5% DMSO, (2) CDD-0102A 1.2 mg/kg/saline with 5% dimethyl sufloxide (DMSO), (3) saline/VU0255035 3 mg/kg, and (4) CDD-0102A 1.2 mg/kg/VU0255035 3 mg/kg (n = 6 per group). VU0255035 was dissolved in saline containing 5% DMSO (Rahman et al., 2020). Each mouse received 2 i.p. injections approximately 15 seconds apart and tested for self-grooming 20 minutes post-injection. Third, in a chronic treatment study, each B6 and BTBR mouse was assigned to 1 of 2 treatment groups: (1) saline or (2) CDD-0102A 1.2 mg/kg. Each mouse received the treatment as an i.p. injection for 14 consecutive days (n = 7–8 per group). This injection procedure was based on previous studies investigating the effects of chronic drug treatment on behavior (Uehara et al., 2013; Dunn et al., 2020). Twenty-four hours after the last injection, each mouse received a self-grooming test.

#### Effect of CDD-0102A in Nesting Removal Test

The test was conducted in each mouse’s home cage, which was a clear plastic cage (28 cm wide × 17 cm long × 12 cm high). Each cage contained approximately 120 g of Sani-chips bedding (Teklad 7090, Madison, WI, USA) and 15 g of crinkle-cut paper nesting material (The Andersons, Maumee, OH, USA). Sani-chips remained in the home cage throughout the test. A camera was mounted above the home cage connected to a computer that ran ANY-maze software, which recorded distance traveled during the test. The time spent digging in the bedding was measured first with nesting material in the home cage (10-minute test) and subsequently with the nesting material removed from the home cage (10-minute test; see supplementary Figure 2). Digging was defined as the repetitive use of front and/or back paws to displace bedding (Lacivita et al., 2020). A trained observer recorded the cumulative amount of time spent digging in each test condition.

Three experiments were conducted. In an acute treatment study, each B6 and BTBR mouse was assigned to 1 of the following 3 groups: (1) saline, (2) CDD-0012A 1.2 mg/kg, or (3) CDD-0102A 3 mg/kg. Separate mice were used for each treatment condition such that a mouse received only a single treatment (n = 8 per group). Twenty minutes after an i.p. injection, the nesting removal test began as described above. In a chronic treatment study, each BTBR and B6 mouse was assigned to 1 of 2 treatment groups: (1) saline, or (2) CDD-0102A 1.2 mg/kg. Each mouse received the assigned treatment as an i.p. injection for 14 consecutive days (n = 8 per group). Twenty-four hours after the last injection, each mouse received the nesting removal test. Third, a study determined whether direct injections of CDD-0102A into the dorsal striatum affected digging in nesting removal test. Seven- to 9-week-old mice received stereotaxic surgery to bilaterally implant cannulae aimed at the dorsomedial striatum. Before surgery, each mouse received an i.p. injection of ketamine (100 mg/kg) and xylazine (10 mg/kg). Two 5-mm stainless-steel guide cannulae (Plastics One, Roanoke, VA, USA) were implanted. The stereotaxic coordinates for the dorsomedial striatum were the following: 1.3 mm anterior to bregma, ±1.1 mm lateral, 1.5 mm below the skull. To minimize pain or discomfort, mice received subcutaneous administration of meloxicam after surgery and for 2 days subsequently. All mice survived surgery and were allowed at least 1 week to recover prior to the start of microinfusions and testing. For the microinfusion, a mouse was placed in a tapered plastic cone that allowed the guide cannulae to protrude out. A 33-gauge injection cannula was inserted into each guide cannula. The injection cannula extended 1 mm beyond the guide cannula tip. The injection cannulae were attached to polyethylene tubes (PE-20) connected to separate 10-μL syringes. A microinfusion pump drove the syringes with solutions infused in a volume of 0.2 μL per side for 2 minutes. The total volume infused was 0.2 μL per side. The cannulae were left in place for 30 seconds to allow drug diffusion around the injector tip. After removal of the injection cannulae, mice were removed from the plastic cone and left undisturbed in their home cage for 1 minute. Subsequently, testing commenced.

Each BTBR and B6 mouse received 3 separate injections and nesting removal tests. Each mouse received the following microinfusions: (1) saline, (2) CDD-0102A 0.1 μg/side, and (3) CDD-0102A 1 μg/side (n = 7–9). A mouse was pseudo-randomly assigned to 1 of 6 injection order combinations to control for order across strain and sex. Successive tests for a given mouse occurred 3–5 days following the previous test.

### Statistical Analysis

Two-way ANOVA was conducted to determine if there was a significant difference between strain and treatment in mice for spatial reversal and grooming studies. For nesting removal, a 3-way ANOVA with repeated measures was conducted to determine if there was a significant difference for strain, treatment, and/or test condition. Post-hoc Tukey multiple comparisons tests were used to determine significant differences between specific groups.

## RESULTS

### Effect of CDD-0102A on Spatial Reversal Learning

**Figure 1.**
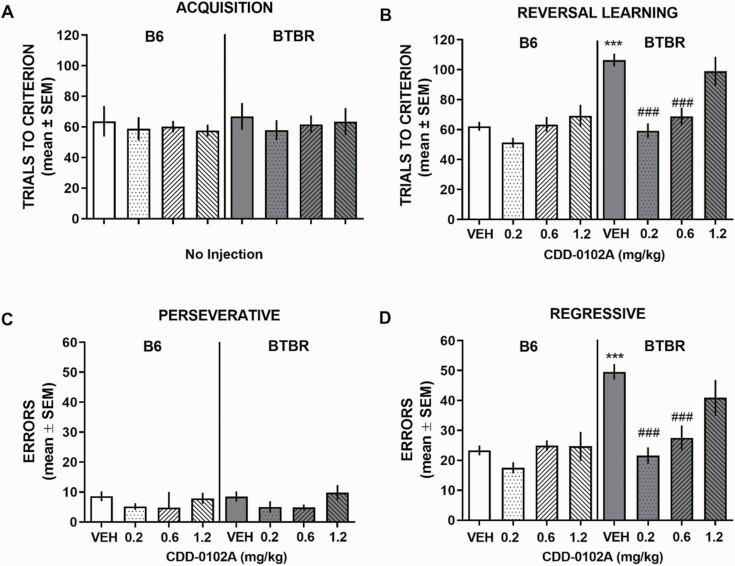
5-(3-ethyl-1,2,4-oxadiazol-5-yl)-1,4,5,6-tetrahydropyrimidine hydrochloride (CDD-0102A) attenuates a probabilistic reversal learning deficit in BTBR mice. Each mouse received a single i.p. injection of either saline (VEH) or 0.2, 0.6, or 1.2 mg/kg of CDD-0102A 30 minutes prior to testing (n = 12–13 per group). (A) B6 and BTBR mice exhibit similar acquisition performance in the number of trials to criterion on a spatial discrimination with probabilistic reinforcement. (B) BTBR-VEH–injected mice required significantly more trials to achieve criterion during probabilistic reversal learning compared with B6-VEH–injected mice. CDD-0102A treatment attenuated the reversal learning deficits in BTBR mice. (C) In reversal learning, there was no strain difference in perseverative errors, although the 0.2- and 0.6-mg/kg doses reduced perseverative errors in BTBR mice. (D) BTBR-VEH–injected mice exhibited a significant increase in regressive errors during reversal learning compared with B6 VEH-injected mice that was significantly attenuated by CDD-0012A in a dose-dependent manner. ***P < .0001 vs B6-VEH, ###*P* < .0001 vs BTBR-VEH.

All groups achieved acquisition criterion in approximately 60 trials (see Figure 1). The reversal learning analysis revealed significant main effects for strain and treatment as well as a significant strain × treatment interaction (*F*_3,89_ = 4.92, *P = *.003). A post-hoc test indicated that BTBR vehicle-treated mice were significantly impaired compared with B6 vehicle-treated mice (*P < *.001). Administration of 0.2 and 0.6, but not 1.2, mg/kg of CDD-0102A significantly reduced trials to criterion compared with that of vehicle-treated BTBR mice (*P < *.0001) and to a level not significantly different than that of vehicle-treated B6 mice (*P* > .05). In B6 mice, CDD-0102A treatment at all doses did not affect reversal learning compared with vehicle treatment. Further, CDD-0102A treatment did not affect trials per minute in either B6 or BTBR mice (see supplementary Figure 3).

Analysis of the perseverative errors (see Figure 1C) indicated there was no significant strain effect or interaction, but a significant treatment effect (*F*_3,89_ = 3.29, *P < *.024) reflecting that the 0.2- and 0.6-mg/kg doses reduced perseverative errors in both strains. For regressive errors (see Figure 1D), there was a significant effect for strain and treatment as well as a significant interaction (*F*_3,89_ = 4.92, *P < *.003). Vehicle-injected BTBR mice committed significantly more regressive errors than vehicle-injected B6 mice (*P* < .0001). CDD-0102A treatment at 0.2 and 0.6, but not 1.2, mg/kg of CDD-0102A significantly reduced regressive errors compared with vehicle-treated BTBR mice (*P < *.0001 and *P* = .0006, respectively) and to a level that did not significantly differ from vehicle-treated B6 mice (*P* > .05).

### Effect of CDD-0102A on Self-Grooming

**Figure 2.**
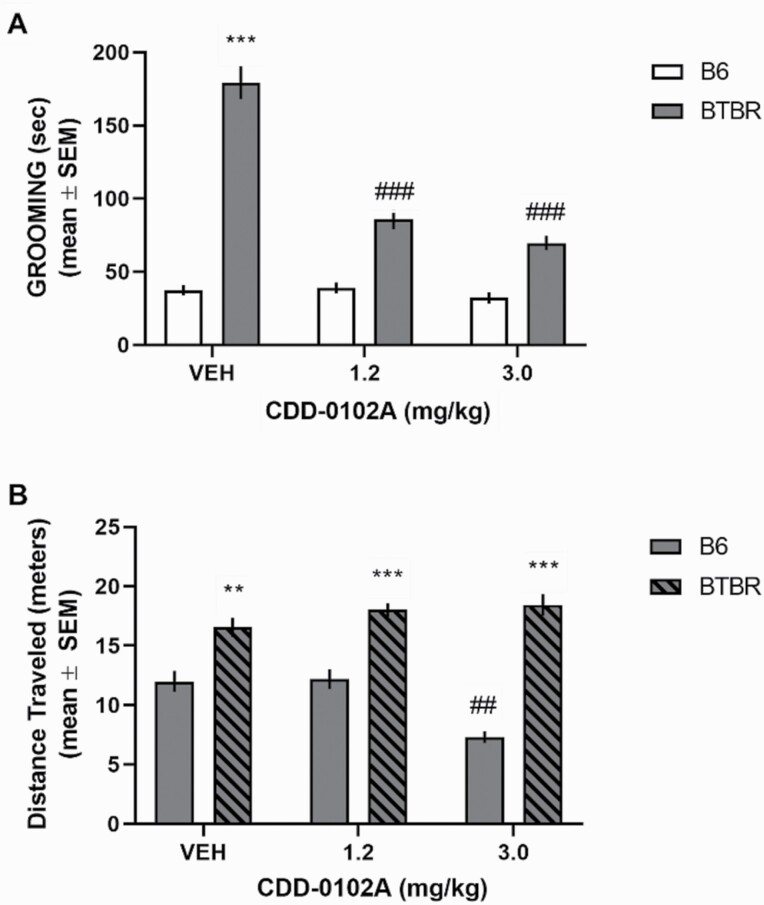
Acute 5-(3-ethyl-1,2,4-oxadiazol-5-yl)-1,4,5,6-tetrahydropyrimidine hydrochloride (CDD-0102A) treatment reduces elevated self-grooming behavior in BTBR mice. Each mouse received a single i.p. injection of either saline (VEH) or 1.2 or 3 mg/kg of CDD-0102A 20 minutes prior to testing (n = 7–8 per group). (A) BTBR-VEH mice exhibit a significant increase in grooming behavior compared with B6 mice. CDD-0102A treatment significantly reduced grooming behavior in BTBR mice. ***P < .0001 vs B6-VEH, ###*P < *.0001 vs BTBR-VEH. (B) BTBR mice exhibited significantly greater distance traveled than B6 mice during the 10-minute self-grooming test. The highest dose of CDD-0102A significantly reduced distance traveled in B6 mice. **P < .001 vs B6 treatment groups, ***P < .0001 vs B6 treatment groups, ###*P < *.0001 vs B6-VEH.

CDD-0102A treatment also reduced grooming in BTBR mice (see Figure 2A). With acute treatment, there was a significant main effect for strain and treatment as well as a significant interaction (*F*_2,40_ = 43.87, *P* < .0001). Vehicle-treated BTBR mice spent significantly more time grooming compared with vehicle-treated B6 mice (*P < *.0001). CDD-0102A treatment in BTBR mice significantly reduced grooming behavior at both doses compared with vehicle-treated BTBR mice (*P < *.0001). BTBR mice exhibited significantly greater locomotor activity than B6 mice during the self-grooming test (see Figure 2B). Analyses of locomotor activity revealed significant main effects for strain and treatment as well as a significant interaction (*F*_2,40_ = 10.56, *P* = .0002). Post-hoc tests revealed that CDD-0102A 3 mg/kg in B6 mice significantly reduced activity compared with the other B6 groups (*P < *.01).

**Figure 3.**
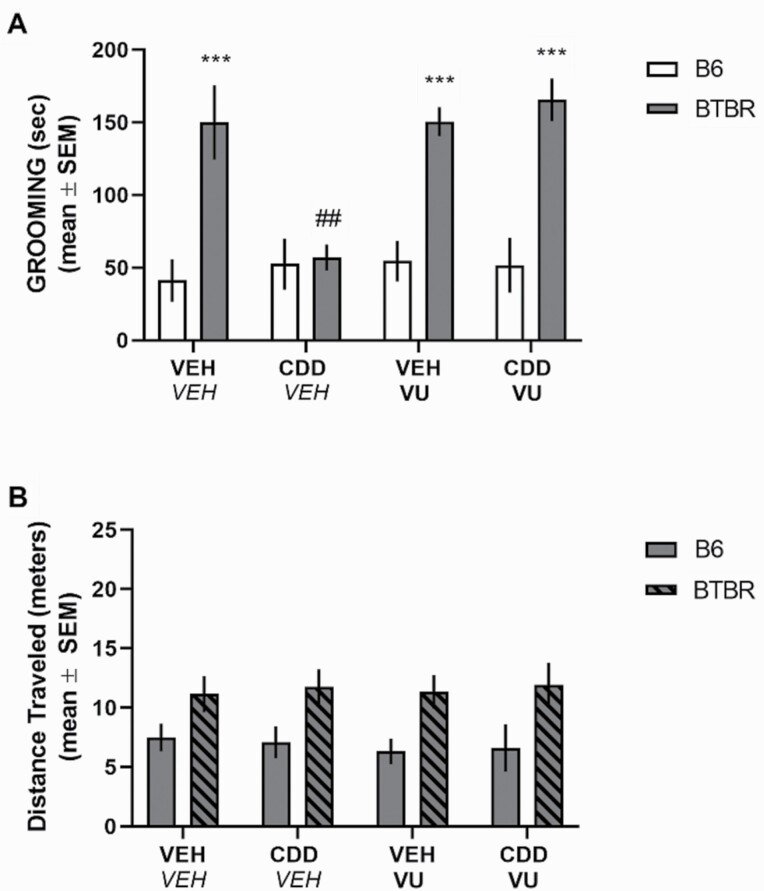
The M_1_ muscarinic antagonist VU 0255035 reversed 5-(3-ethyl-1,2,4-oxadiazol-5-yl)-1,4,5,6-tetrahydropyrimidine hydrochloride (CDD-0102A)–induced reduction of self-grooming behavior in BTBR mice. Each mouse received 2 i.p. injections approximately 15 seconds apart. Mice were tested 20 minutes post-injection. One injection was either saline with 5% DMSO (*VEH*) or 3 mg/kg VU 0255035 (VU) followed by a second injection of saline (VEH) or 1.2 mg/kg of CDD-0102A (CDD) (n = 6 per group). (A) BTBR-VEH mice exhibited a significant increase in grooming behavior compared with B6 mice. CDD-0102A treatment significantly reduced grooming behavior in BTBR mice. VU 0255035 treatment significantly reversed the CDD-0102A-induced reduction in grooming behavior in BTBR mice. ***P < .0001 vs B6 treatment groups, ###*P < *.0001 vs BTBR-VEH. (B) BTBR mice exhibited greater distance traveled than B6 mice during 10-minute self-grooming test.

Analysis of VU0255035 alone and in combination with CDD-0102A (see Figure 3) indicated a significant effect for strain and treatment as well as a significant interaction (*F*_3,40_ = 4.97, *P* = .0051). CDD-0102A 1.2 mg/kg significantly reduced grooming behavior compared with vehicle-treated BTBR mice (*P < *.0001). VU0255035 3 mg/kg treatment in BTBR mice did not affect grooming compared with vehicle-injected BTBR mice (*P* > .05). However, combined VU 0255035/CDD-0102A treatment in BTBR mice significantly elevated grooming duration compared with CDD-0102A treatment alone (*P* < .001) and to a level comparable with vehicle-treated BTBR mice (*P* > .05). Analyses of locomotion during grooming indicated only a significant effect for strain (*F*_1,40_ = 18.86, *P* < .0001), reflecting that BTBR mice had greater locomotor activity than B6 mice.

**Figure 4.**
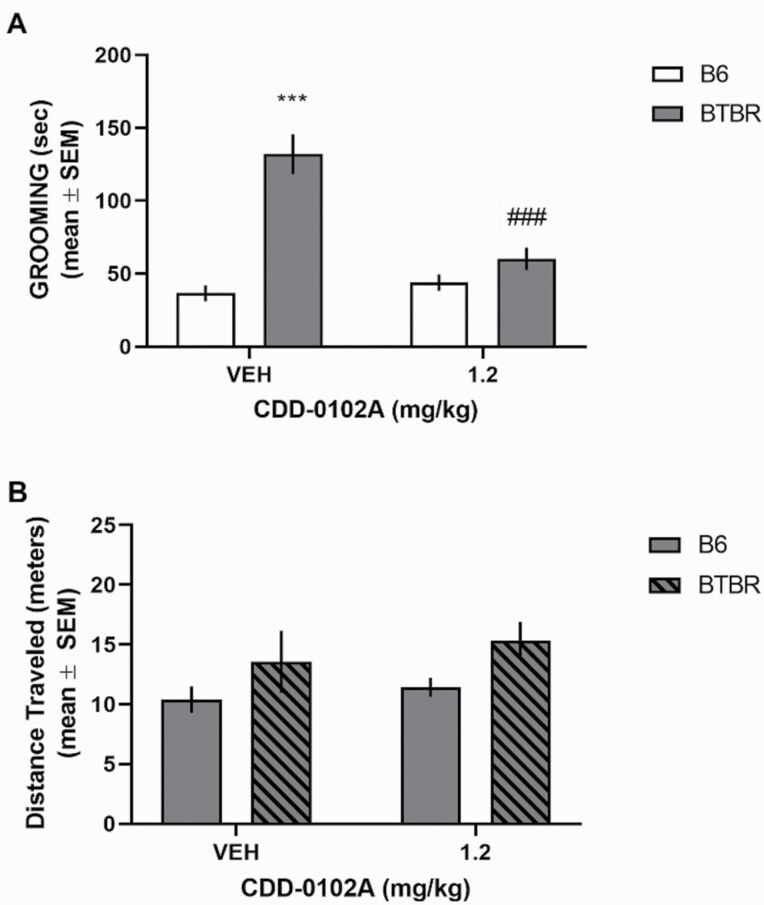
Chronic 5-(3-ethyl-1,2,4-oxadiazol-5-yl)-1,4,5,6-tetrahydropyrimidine hydrochloride (CDD-0102A) treatment significantly reduced elevated self-grooming behavior in BTBR mice. Each mouse received 14 consecutive days of i.p. injections of either saline (VEH) or 1.2 mg/kg of CDD-0102A (n = 6–8 per group). Mice were tested 24 hours after the last injection. (A) BTBR-VEH mice exhibited a significant increase in grooming behavior. ***P < .0001 vs B6-VEH, ###*P < *.0001 vs BTBR-VEH. Fourteen days of CDD-0102A treatment significantly reduced elevated grooming behavior in BTBR mice. (B) BTBR mice exhibited significantly greater distance traveled than B6 mice during the 10 minute self-grooming test. Repeated treatment with CDD-0102A did not affect distance traveled in BTBR mice.

Chronic CDD-0102A treatment decreased grooming in BTBR mice (see Figure 4). There was a significant effect for strain and treatment as well as a significant interaction (*F*_1,25_ = 19.99, *P* = .0001). CDD-0102A 1.2 mg/kg significantly reduced grooming behavior compared with vehicle-treated BTBR mice (*P < *.0001). Analyses of locomotor behavior during grooming indicated there was only a significant effect for strain (*F*_1,25_ = 4.64, *P* = .041), reflecting that BTBR mice had greater locomotor activity than B6 mice.

### Effect of CDD-0102A Treatment in Nesting Removal Test

**Figure 5.**
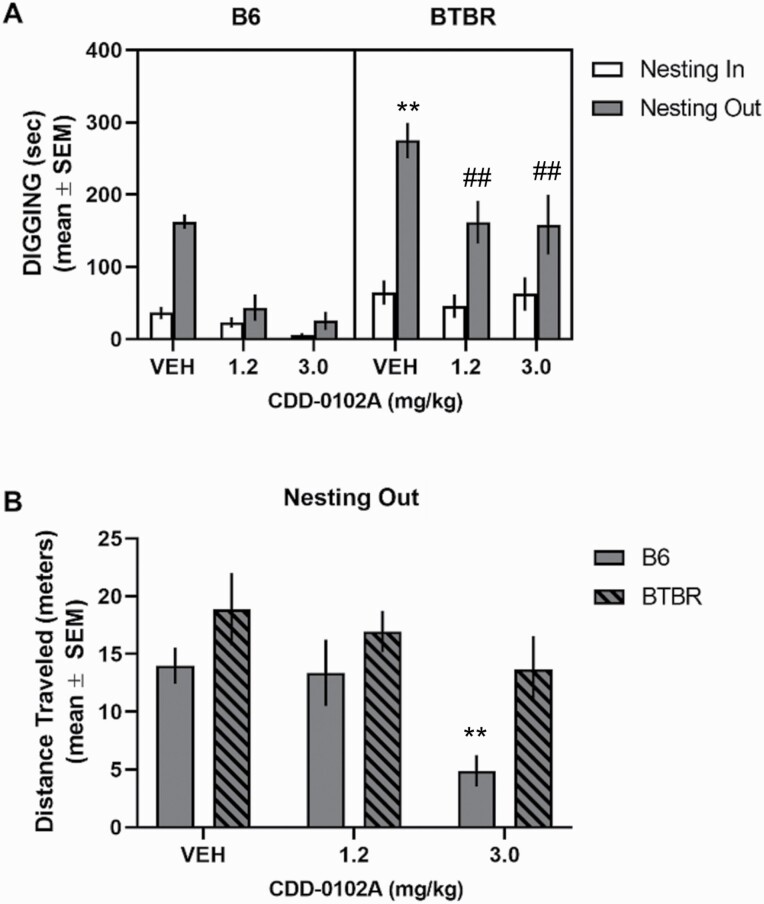
Acute 5-(3-ethyl-1,2,4-oxadiazol-5-yl)-1,4,5,6-tetrahydropyrimidine hydrochloride (CDD-0102A) treatment reduced elevated digging behavior in the nesting removal test. Each mouse received a single i.p. injection of either saline (VEH) or 1.2 or 3 mg/kg of CDD-0102A 20 minutes prior to testing (n = 8 per group). (A) B6 and BTBR mice exhibited an increase in digging behavior with removal of nesting material. CDD-0102A treatment significantly reduced the elevated digging behavior with nesting-out in both B6 and BTBR mice. **P < .001 vs B6-VEH nesting-out, ##*P* < .01 vs BTBR-VEH nesting-out. (B) BTBR mice exhibited greater distance traveled during the 10-minute nesting out condition compared with B6 mice. The highest dose of CDD-0102A reduced distanced traveled. **P < .001 vs B6 VEH nesting out.

Removal of nesting material significantly increased digging in vehicle-injected B6 mice (*t*_7_ = 12.47, *P* < .0001) and vehicle-injected BTBR mice (*t*_7_ = 7.74, *P* < .0001) (see Figure 5). However, the magnitude change in digging behavior from the nesting-in to the nesting-out condition was significantly greater in BTBR mice compared with B6 mice (*t*_14_ = 2.91, *P* = .01). For acute treatment, there were significant main effects for strain, treatment, and condition. There was a significant treatment × condition interaction (F_2,42_ = 10.61, *P* = .0002), reflecting that CDD-0102A treatment at both doses reduced digging in both strains during the nesting-out condition. There was also a significant strain × condition interaction (*F*_1,42_ = 15.67, *P* = .0003), reflecting that both doses of CDD-0102A treatment in B6 mice reduced digging in the nesting-out condition to a level similar to that of the nesting-in condition. In contrast, both doses of CDD-0102A treatment also reduced digging behavior in BTBR mice during the nesting-out condition but to levels that were still higher than in the nesting-in condition. Analyses of locomotor activity in the nesting-out condition revealed a significant main effect for strain (*F*_1,42_ = 8.77, *P = *.005). Acute treatment with CDD-0102A at the 3-mg/kg dose reduced locomotor activity in both strains, resulting in a significant effect for treatment (*F*_2, 42_ = 5.11, *P = *.01).

**Figure 6.**
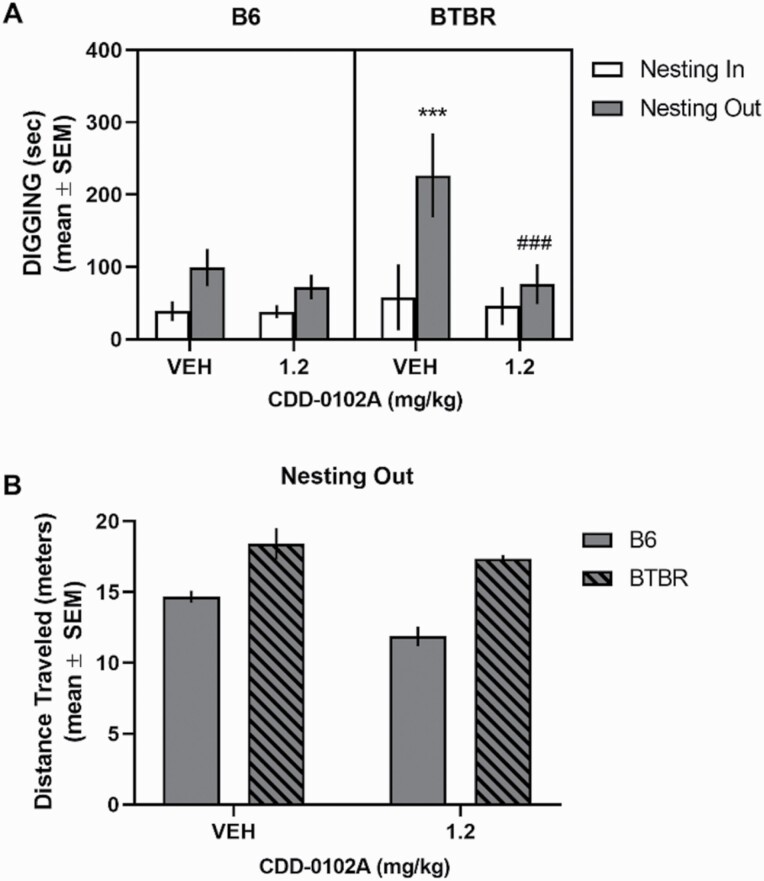
Chronic 5-(3-ethyl-1,2,4-oxadiazol-5-yl)-1,4,5,6-tetrahydropyrimidine hydrochloride (CDD-0102A) treatment reduced elevated digging behavior in the nesting removal test in BTBR mice. Each mouse received 14 consecutive days of i.p. injections of either saline (VEH) or 1.2 mg/kg of CDD-0102A (n = 8 per group). Mice were tested 24 hours after the last injection. (A) Digging behavior in nesting removal test. B6 and BTBR mice exhibited an increase in digging behavior with removal of nesting material. Fourteen days of CDD-0102A treatment significantly reduced the elevated digging behavior with nesting-out in BTBR mice. ***P < .0001 vs BTBR-VEH nesting-in and B6-VEH nesting-out, ###*P < *.0001 vs BTBR-VEH nesting out. (B) BTBR mice exhibited greater distance traveled during nesting-out condition compared with B6 mice. Repeated treatment with CDD-0102A did not affect distance traveled in BTBR mice.

In the chronic treatment study, removal of nesting material significantly increased digging in vehicle-injected B6 mice (*t*_7_ = 10.09, *P* < .0001) and vehicle-injected BTBR mice (*t*_7_ = 13.94, *P* < .0001) (see Figure 6A). The magnitude of change in digging across nesting conditions was significantly greater in BTBR mice compared with B6 mice (*t*_14_ = 8.06, *P* < .0001). A 3-way ANOVA with repeated measures indicated that all main effects were significant as well as 2-way interactions. There was also a significant strain × treatment × condition interaction (F_1,28_ = 46.95, *P* < .0001). Post-hoc analyses revealed that vehicle-injected BTBR mice exhibited a significantly greater amount of digging in the nesting-out vs nesting-in condition (*P < *.0001) and compared with the nesting-out condition of B6 mice (*P < *.0001). Further, in the nesting-out condition, CDD-0102A treatment in BTBR mice significantly reduced digging compared with vehicle-treated BTBR mice (*P < *.0001) and to a level similar to vehicle-treated B6 mice (*P* > .05). In contrast, CDD-0102A treatment in B6 mice did not significantly reduce digging compared with vehicle treatment in B6 mice in the nesting-out condition (*P* > .05). Analysis of locomotor activity in the nesting-out condition (see Figure 6B) indicated that BTBR mice exhibited significantly greater locomotor activity than B6 mice (*F*_1,28_ = 43.20, *P < *.0001). There was also a significant treatment effect (*F*_1, 28_ = 7.59, *P = *.01) because CDD-0102A treatment tended to reduce locomotor activity in both strains. However, post-hoc analyses revealed that CDD-0102A treatment in B6 mice, but not BTBR mice, significantly reduced locomotor activity compared with vehicle treatment (*P* < .05).

To determine whether CDD-0102A may, at least in part, act in the dorsal striatum to reduce stereotyped motor behaviors, the effects of the drug injected into the dorsomedial striatum on digging behavior were investigated.

**Figure 7.**
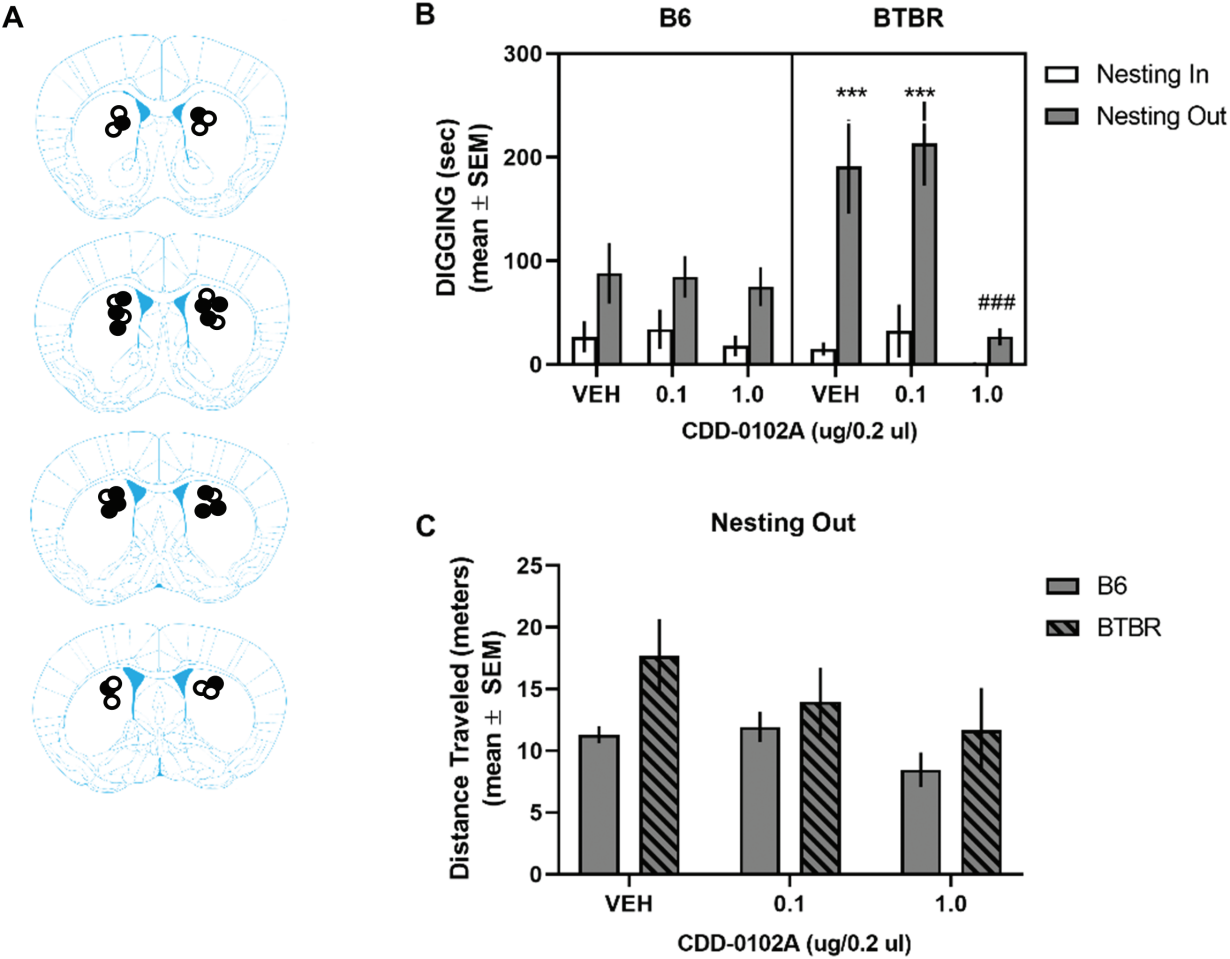
5-(3-ethyl-1,2,4-oxadiazol-5-yl)-1,4,5,6-tetrahydropyrimidine hydrochloride (CDD-0102A) infusion into the dorsal striatum attenuated elevated digging behavior in the nesting removal test in BTBR mice. Each mouse received a single, bilateral intracranial injection of saline (VEH) or 0.1 and 1.0 μg/0.2 μL of CDD-0102A 5 minutes prior to testing (n = 7–9 per group) across multiple test sessions. (A) Cannula tip placements in the dorsal striatum of B6 and BTBR mice included in the behavioral analyses. Mouse brain sections adapted from The Mouse Brain in Stereotaxic Coordinates (Paxinos and Franklin 2001). ***P < .0001 vs BTBR-VEH nesting-in and B6-VEH nesting-out, ###*P < *.0001 vs BTBR-VEH nesting-out. (B) Intrastriatal infusion of CDD-0102A into the dorsal striatum of BTBR mice significantly attenuated elevated digging behavior in the nesting removal test. (C) BTBR mice exhibited significantly greater distance traveled during nesting-out condition compared with B6 mice. Intrastriatal injection of CDD-0102A did not affect distance traveled in BTBR mice. ●, cannula placements for BTBR mice; ○, cannula placements for B6 mice.

Histological analysis indicated that cannula placements were principally located in the dorsomedial striatum at the level of the genu of the corpus callosum (Figure 7A).

Again, removal of nesting material significantly increased digging in vehicle-injected B6 mice (*t*_6_ = 3.19, *P* = .019) and vehicle-injected BTBR mice (*t*_7_ = 4.31, *P* < .0035) (see Figure 7B), with a significantly greater magnitude change in vehicle-treated BTBR mice compared with B6 mice (*t*_*13*_ = 2.43, *P* = .03). A 3-way ANOVA with repeated measures revealed that there were significant main effects for treatment and condition, but not strain. The analysis further showed that there was a significant strain × treatment × condition interaction (*F*_2,39_ = 6.57, *P* = .0035). Post-hoc tests indicated that vehicle-treated BTBR mice spent significantly more time digging in the nesting-out vs nesting-in condition (*P* < .0001). BTBR mice injected with 1 μg/side, but not 0.1 μg/side, of CDD-0102A spent significantly less time digging in the nesting-out condition compared with vehicle-treated BTBR mice (*P = *.0003) and to a level similar to that of vehicle-treated B6 mice in the nesting-out condition (*P* > .05). In contrast, CDD-0102A treatment, at both doses, in B6 mice did not significantly reduce digging behavior compared with vehicle-treated B6 mice in the nesting-out condition (*P* > .05). Analysis of locomotor activity in the nesting-out condition (see Figure 7C) indicated that, overall, BTBR mice tended to locomote more than B6 mice as reflected by a main effect of strain that approached significance (*F*_1, 39_ = 3.81, *P* = .06).

**Figure 8.**
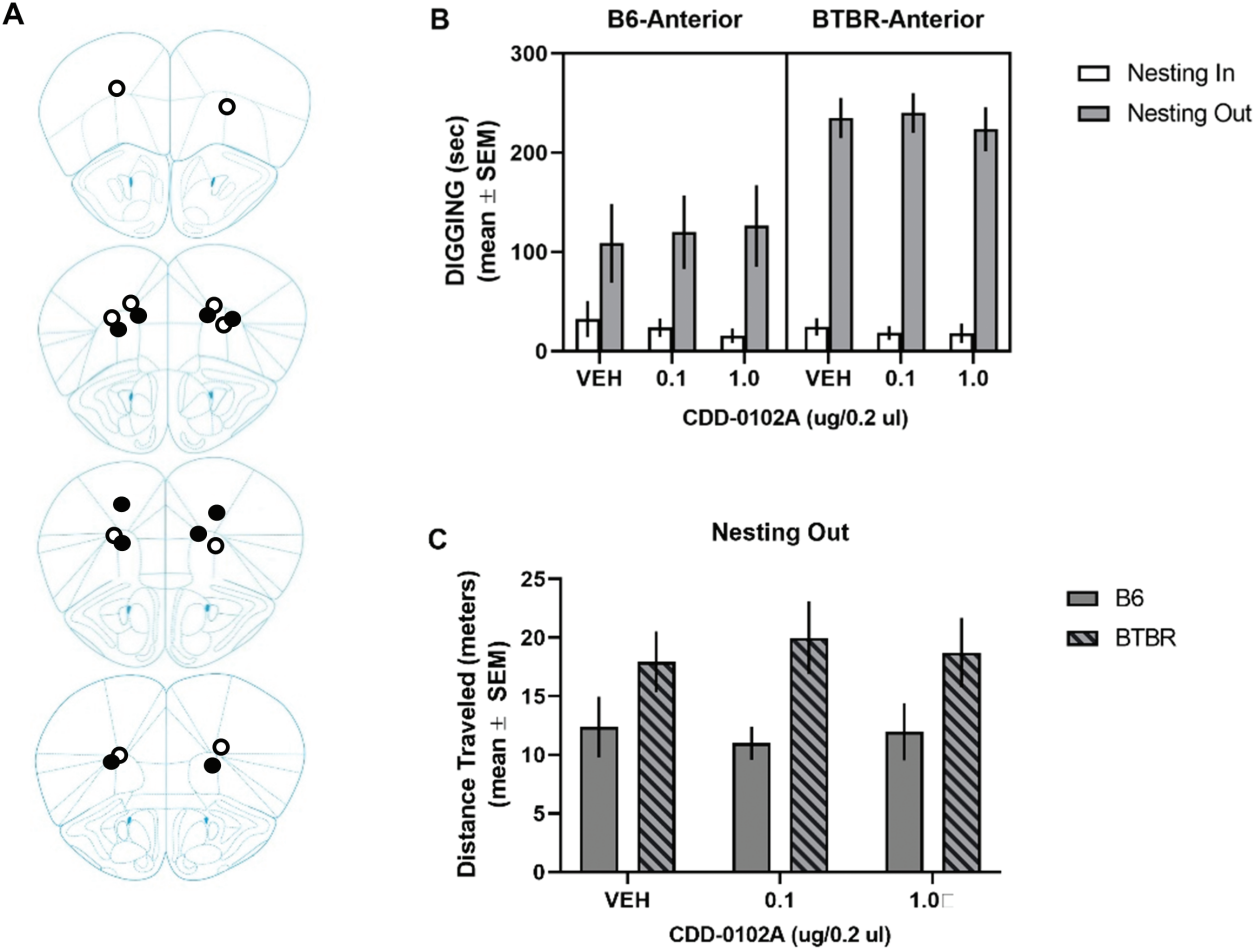
5-(3-ethyl-1,2,4-oxadiazol-5-yl)-1,4,5,6-tetrahydropyrimidine hydrochloride (CDD-0102A) infusion into the frontal cortex did not affect elevated digging behavior in the nesting removal test. Each mouse received a single, bilateral intracranial injection of saline (VEH) or 0.1 and 1.0 μg/0.2 μL of CDD-0102A 5 minutes prior to testing (n = 5 per group) across multiple test sessions. (A) Cannula tip placements in the frontal cortex of B6 and BTBR mice included in the behavioral analyses. Mouse brain sections adapted from The Mouse Brain in Stereotaxic Coordinates (Paxinos and Franklin 2001). (B) Intrastriatal infusion of CDD-0102A into the frontal cortex did not affect digging behavior in B6 or BTBR mice. (C) BTBR mice exhibit significantly greater distance traveled during nesting-out condition compared with B6 mice. Intrastriatal injection of CDD-0102A did not affect distance traveled. ●, cannula placements for BTBR mice; ○, cannula placements for B6 mice.

Several mice from each strain (n = 5 per strain) had bilateral cannula placements that were anterior to the dorsomedial striatum located in the frontal cortex (see Figure 8). An analysis conducted on digging behavior revealed that there was no significant effect for treatment, but there were significant main effects for strain and condition. The only significant interaction was the strain × condition (*F*_1, 23_ = 30.49, *P* < .0001), reflecting that BTBR mice exhibited greater digging behavior compared with B6 mice in the nesting-out condition across all treatments. Thus, CDD-0102A infusions anterior to the dorsomedial striatum did not affect digging behavior in either BTBR or B6 mice. BTBR mice showed greater locomotor activity than B6 mice in the nesting-out condition. This is reflected in a significant main effect for strain (*F*_1, 24_ = 11.11, *P* = .003).

## Discussion

The results indicate that the partial M_1_ muscarinic receptor agonist CDD-0102A alleviates a behavioral flexibility deficit and elevated stereotyped motor behaviors in BTBR mice. The behavioral flexibility deficit displayed as a probabilistic reversal learning impairment. As observed previously in BTBR mice and ASD individuals (D’Cruz et al., 2013; Amodeo et al., 2017, 2018), the reversal learning deficit resulted from an increase in regressive errors that was significantly attenuated by CDD-0102A treatment. Although there was no difference in the number of perseverative errors between the 2 strains, CDD-0102A treatment did reduce perseverative errors in both B6 and BTBR mice. This is similar to that observed in Long-Evans rats in which the drug reduced perseveration in a set-shifting test (Ragozzino et al., 2012). Because there can be significant heterogeneity in the phenotype among ASD individuals, including in cognitive flexibility (Demetriou et al., 2019), CDD-0102A treatment may also be effective in ASD individuals who exhibit a perseverative phenotype by being impaired in initially shifting away from a previously learned choice pattern.

Comparable with reversal learning, a CDD-0102A injection administered acutely or chronically significantly reduced grooming behavior in BTBR mice. The doses used were higher than those used as in reversal learning. Because mice were under food restriction for reversal learning, but not self-grooming, different effective doses were found on these 2 tests as observed in past studies (Amodeo et al., 2018; Dunn et al., 2020). The drug effect did not affect overall locomotor activity. In addition, the selective M_1_ muscarinic receptor antagonist VU 0255035 blocked the effect of CDD-0102A on grooming in BTBR mice, suggesting that the effect of CDD-0102A may be acting principally through M_1_ muscarinic receptors.

In the nesting removal test, both mouse strains exhibited an increase in digging following removal of the nesting. However, a significantly greater magnitude increase occurred in BTBR mice. Acute CDD-0102A treatment significantly reduced digging in both strains. However, only the 1.2-mg dose reduced digging behavior without a more general effect on locomotor activity. Further, chronic treatment with CDD-0012A at 1.2 mg/kg attenuated digging in BTBR, but not B6 mice. One possibility is that different doses of CDD-0102A with chronic treatment may reduce digging in B6 mice. Future studies can address this possibility. The findings from self-grooming and nesting removal tests indicate that CDD-0102A treatment is not only effective in alleviating behavioral flexibility deficits in a mouse model of autism but can also ameliorate elevated stereotyped motor behaviors both acute and chronic administration. Thus, treatment with a partial M_1_ muscarinic agonist such as CDD-0102A may be effective in treating a range of RRBs in autism.

Because the striatum has shown anatomical changes related to RRBs in ASD (Hollander et al., 2005; Langen et al., 2014) and altered striatal functioning in rodent models of autism is related to RRBs (Lewis et al., 2007; Jaramillo et al., 2017; Shonesy et al., 2018), we examined whether direct infusions of CDD-0102A into the striatum affected digging in the nesting removal test. CDD-0102A infused into the dorsal striatum blocked increased digging behavior with nesting removal in BTBR mice. Further, there appeared to be some anatomical selectivity for the effect because infusions rostral to the dorsal striatum did not have a behavioral effect. Thus, despite both the striatum and frontal cortex expressing moderate to high levels of M_1_ muscarinic receptors (Levey et al., 1991, 1995), only a striatal injection was effective in dampening digging behavior. The results suggest that stimulation of M_1_ muscarinic receptors in the dorsal striatum is sufficient to alleviate elevated expression of RRBs when induced by a change in the home environment.

There are multiple mechanisms in striatal circuitry proposed to explain elevated stereotyped motor behaviors and/or behavioral inflexibility (Tanimura et al., 2010; Bloem et al., 2017; Wang et al., 2017; Prager and Plotkin 2019). One possibility is that stimulation of M_1_ muscarinic receptors modulates striatal dopamine signaling to attenuate elevated stereotyped motor behaviors as observed in the nesting removal test. Enhanced dopamine signaling in the striatum particularly by activating dopamine D_1_ receptors leads to stereotyped motor behaviors (Chartoff et al., 2001; Presti et al., 2003; Berridge et al., 2005; Bouchekioua et al., 2018; Lee et al., 2018). Further, removal of M_1_ muscarinic receptors increases striatal dopamine signaling (Gerber et al., 2001), and stimulating M_1_ muscarinic receptors can attenuate motor stereotypies induced by elevating brain dopamine levels (Crittenden et al., 2019). Thus, CDD-0102A treatment may, at least in part, modulate striatal dopamine signaling to attenuate behavioral inflexibility and stereotyped motor behaviors.

Overall, the findings with the partial M_1_ muscarinic agonist CDD-0102A suggest that stimulating M_1_ muscarinic receptors may serve as a novel therapeutic approach to alleviate RRBs in ASD, including both repetitive motor behaviors and cognitive flexibility deficits. This is based on the present results in the BTBR mouse. Support for M_1_ muscarinic receptors as a therapeutic target can be strengthened by examining the effects of CDD-0102A in other mouse models of autism as well as developing other drugs that target M_1_ muscarinic receptors. In recent years, selective positive allosteric modulators of M_1_ muscarinic receptors and M_1_ muscarinic receptor agonists that act at the orthosteric site have been developed (Kruse et al., 2014; Felder et al., 2018; Moran et al., 2019). These drugs have been developed as potential therapeutics to treat Alzheimer’s disease, Lewy body dementia, and schizophrenia (Kruse et al., 2014; Felder et al., 2018; Moran et al., 2019). For example, other partial M_1_ muscarinic agonists such as xanomeline exhibit robust activity in rodent models of schizophrenia as well as in treating symptoms in schizophrenia patients (Stanhope et al., 2001; Shekhar et al., 2008). Because of evidence that altered brain muscarinic receptor signaling occurs in ASD (Perry et al., 2001; Lee et al., 2012) combined with the present findings, treatment with a drug that has high selectivity for M_1_ muscarinic receptors such as CDD-0102A may serve as an effective therapy in alleviating core symptomology in ASD.

## Supplementary Material

pyab079_suppl_Supplementary_Figure_S1

pyab079_suppl_Supplementary_Figure_S2

pyab079_suppl_Supplementary_Figure_S3

## Interest Statement

Dr Messer and Dr Ragozzino share a patent for CDD-102A,[5-(3-ethyl-1, 2, 4-oxadiazol-5-yl)-1, 4, 5, 6-tetra hydropyrimidine) as a cognitive enhancer. US Patent # 10835532.
