# Supplementary figures and images for: Effects of the Partial M1 Muscarinic Cholinergic Receptor Agonist CDD-0102A on Stereotyped Motor Behaviors and Reversal Learning in the BTBR Mouse Model of Autism
Source: Int J Neuropsychopharmacol. 2021 Nov 16;25(1):64–74. doi: 10.1093/ijnp/pyab079 (PMC8756088; doi:10.1093/ijnp/pyab079)

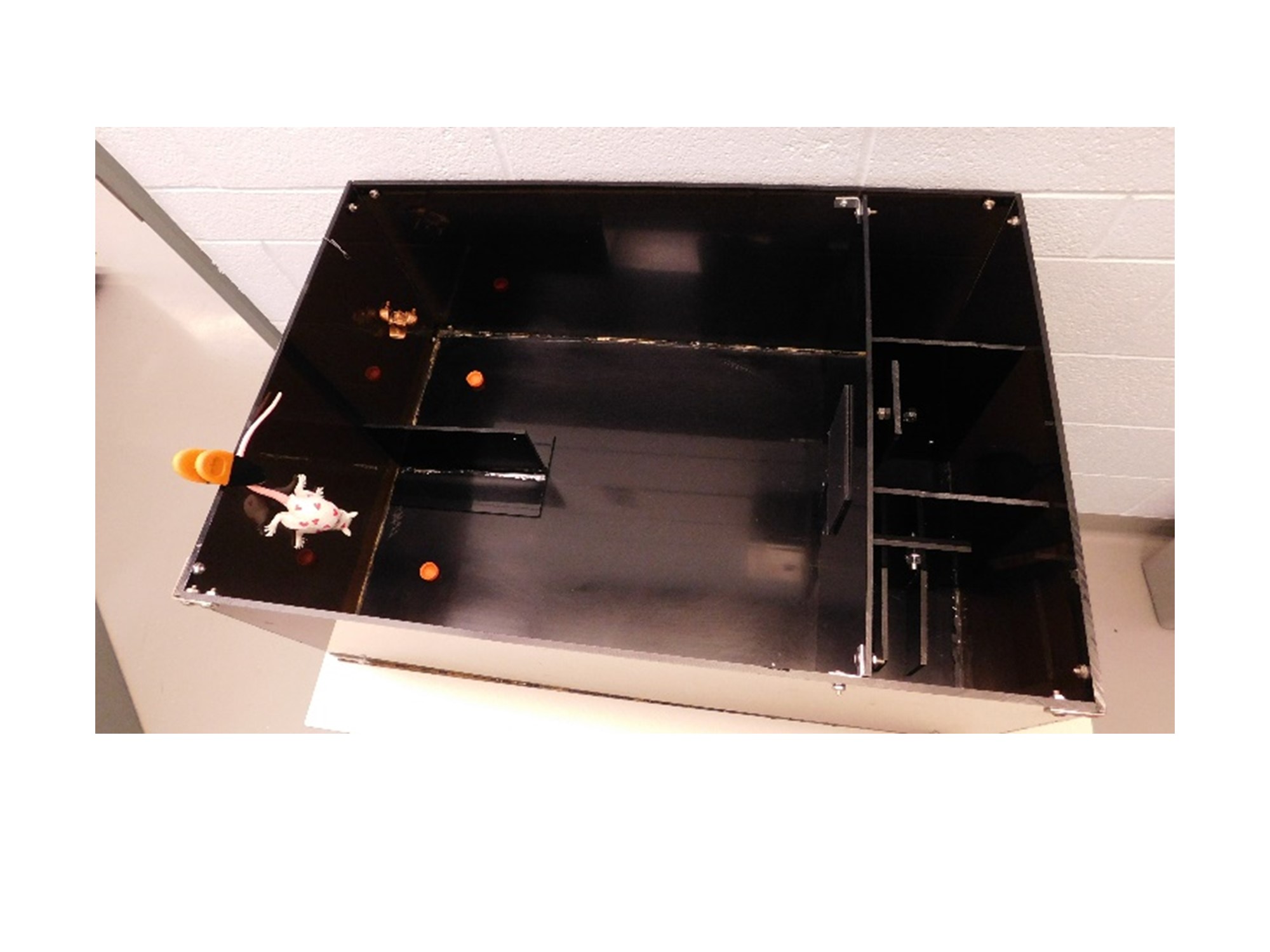

Supplement: pyab079_suppl_Supplementary_Figure_S1 [file pyab079_suppl_supplementary_figure_s1.jpeg]

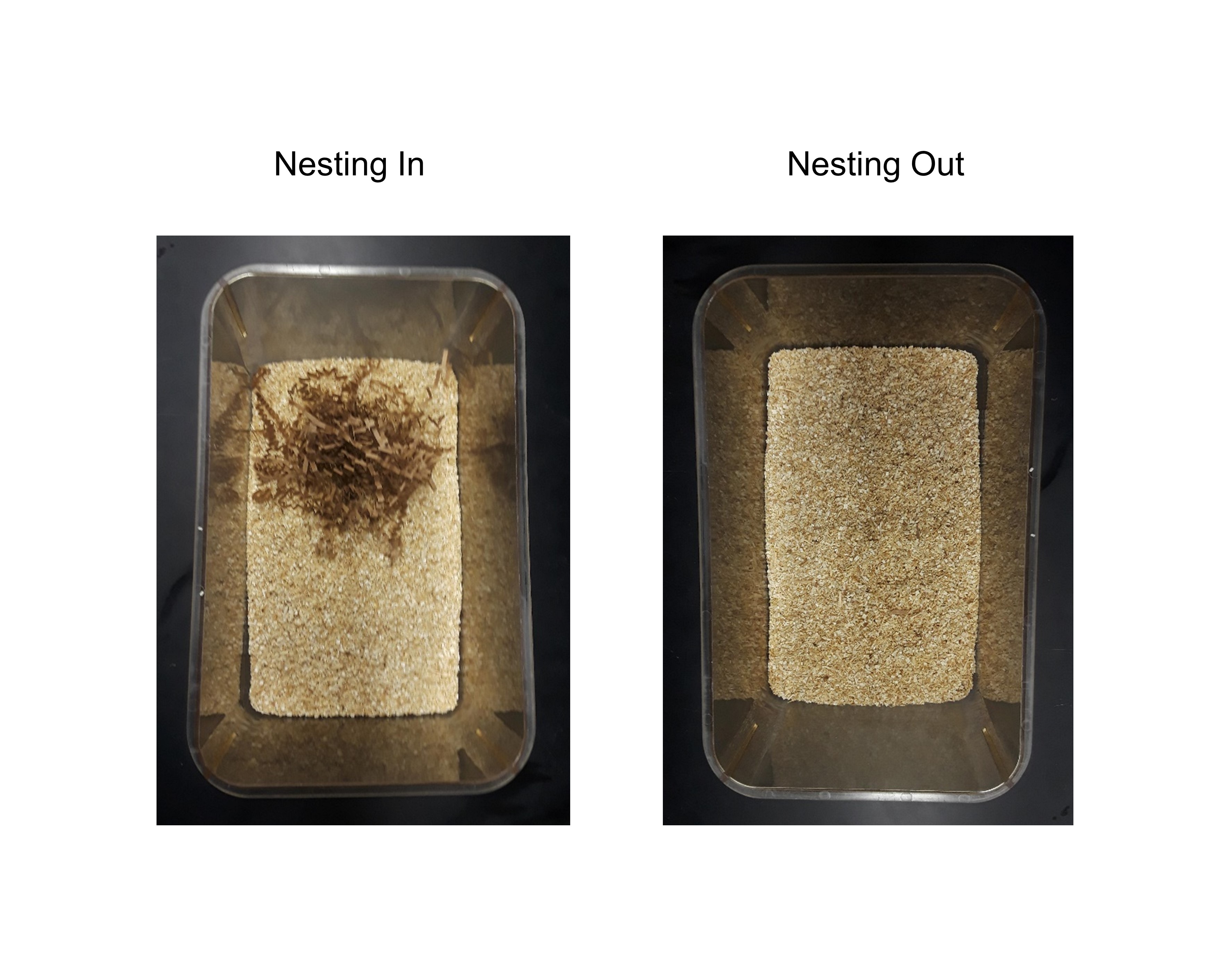

Supplement: pyab079_suppl_Supplementary_Figure_S2 [file pyab079_suppl_supplementary_figure_s2.jpeg]

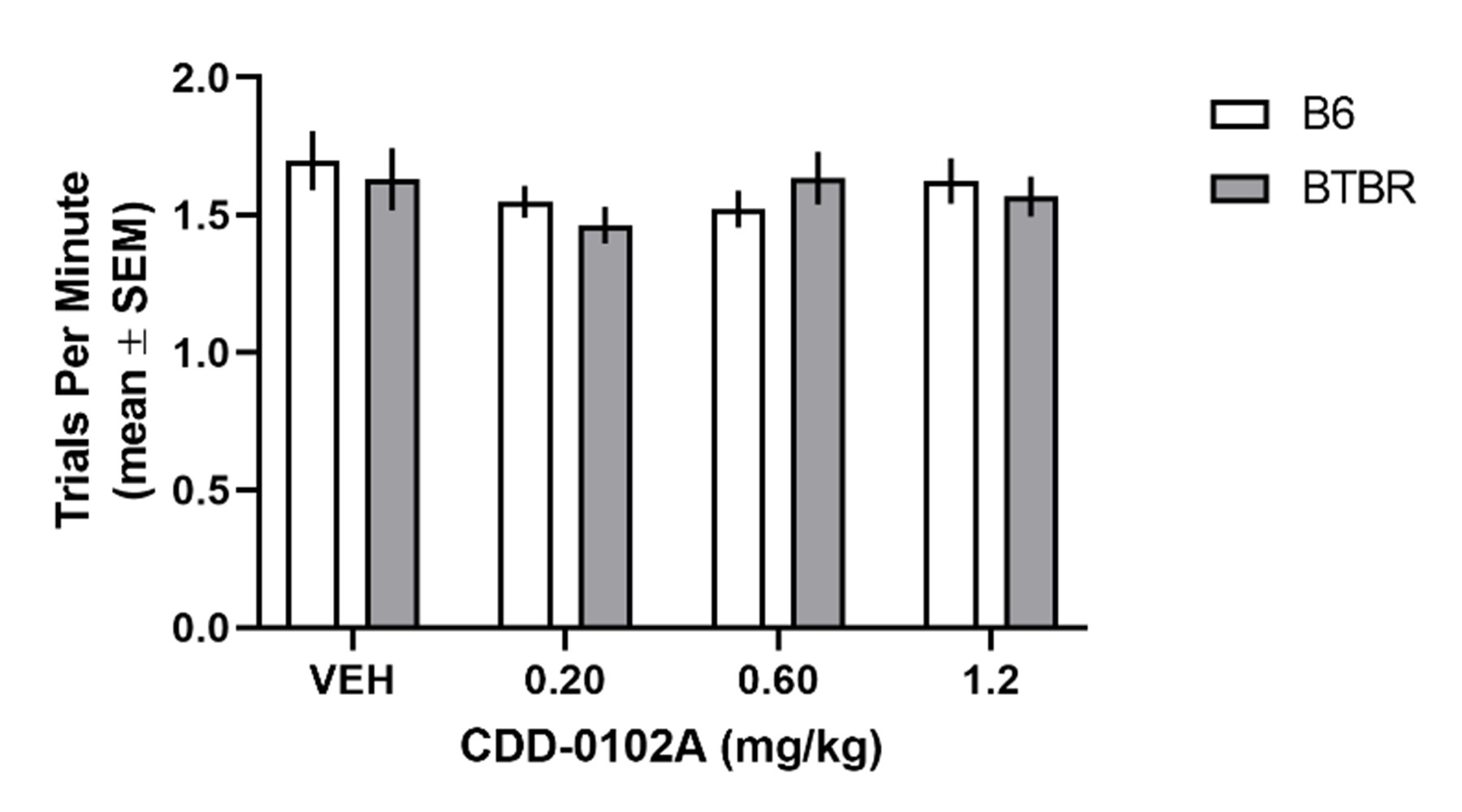

Supplement: pyab079_suppl_Supplementary_Figure_S3 [file pyab079_suppl_supplementary_figure_s3.jpeg]
